# Supplementary material for: A Mild Method for Encapsulation of Citral in Monodispersed Alginate Microcapsules
Source: Polymers (Basel). 2022 Mar 15;14(6):1165. doi: 10.3390/polym14061165 (PMC8954088; doi:10.3390/polym14061165)
Supplement: Supplementary file 1 [file polymers-14-01165-s001.zip › polymers-1622517-supplementary.pdf]

Supporting Information

Table S1. Flow rates for preparation O/W/O emulsions with different sodium alginate concentration in the middle aqueous layers

Concerntration of alginate (w/v)	Flow rates of inner fluid (μL/min)	Flow rates of middle fluid (μL/min)	Flow rates of outer fluid (μL/min)	Thickness of aqueous layer (μm)
1%	2	6	24	38.74
1.5%	2	6	28	39.04
2%	2	6	30	39.33
2.5%	2	6	35	39.73
3%	2	6	50	38.79

Table S2. Recipe of O/W/O emulsions for preparing alginate microcapsules

Fluid	Composition
Inner fluid	SO/BB(V_{SO}:V_{BB}=1:1) + PGPR(2%,w/v)
Middle fliud	Alginate sodium (2%, w/v) + EDTA-Ca (0.1 M) + GDL (2%, w/v) + Pluronic F-127 (0.5%, w/v) +DI water
Outer fluid	SO + PGPR (5%, w/v)
Collection fluid	SO + PGPR (5%, w/v)

Table S3. Fitting results of citral release kinetics from alginate microcapsules

Equation type	Fitted equation	R^2
Zero-order release equation	$Q = 0.00583t + 0.1741$	0.8848
First -order release equation	$Q = 1.2216(1 - e^{-0.011t})$	0.9801
Higuchi plane diffusion equation	$Q = 0.0941t^{1/2} - 0.15$	0.9606
Retger-peppas equation	$Q = 0.0441x^{0.6244}$	0.9515

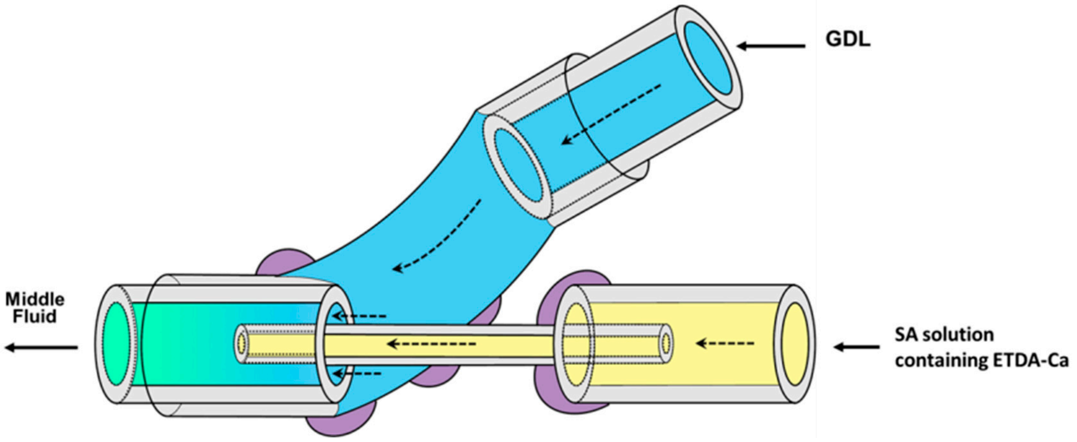

Figure S1. Schematic illustration of Y-shaped micro-mixer.

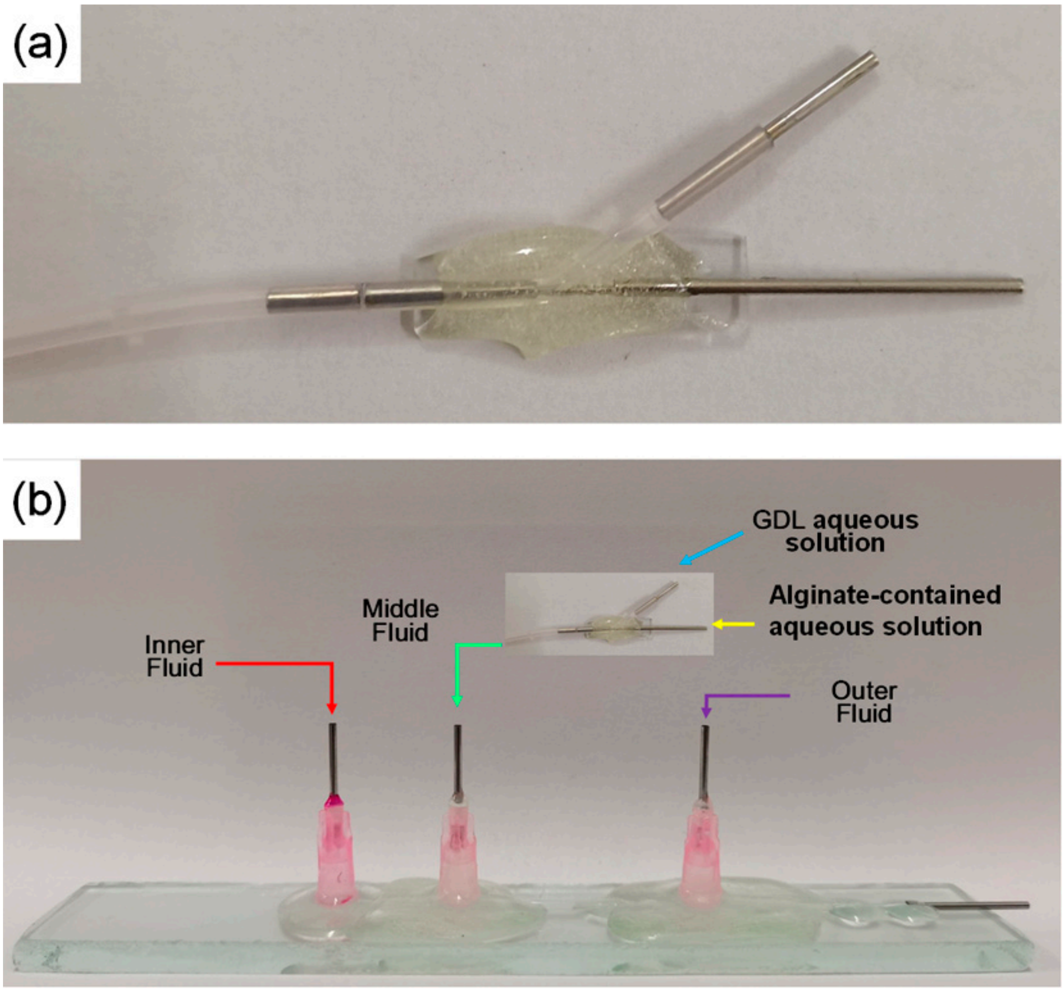

Figure S2. Preparation of O/W/O emulsions with Y-shaped micro-mixer. (**a**) Optical photograph of Y-shaped micro-mixer. (**b**) Connection of the Y-shaped micro-mixer to the microfluidic device.

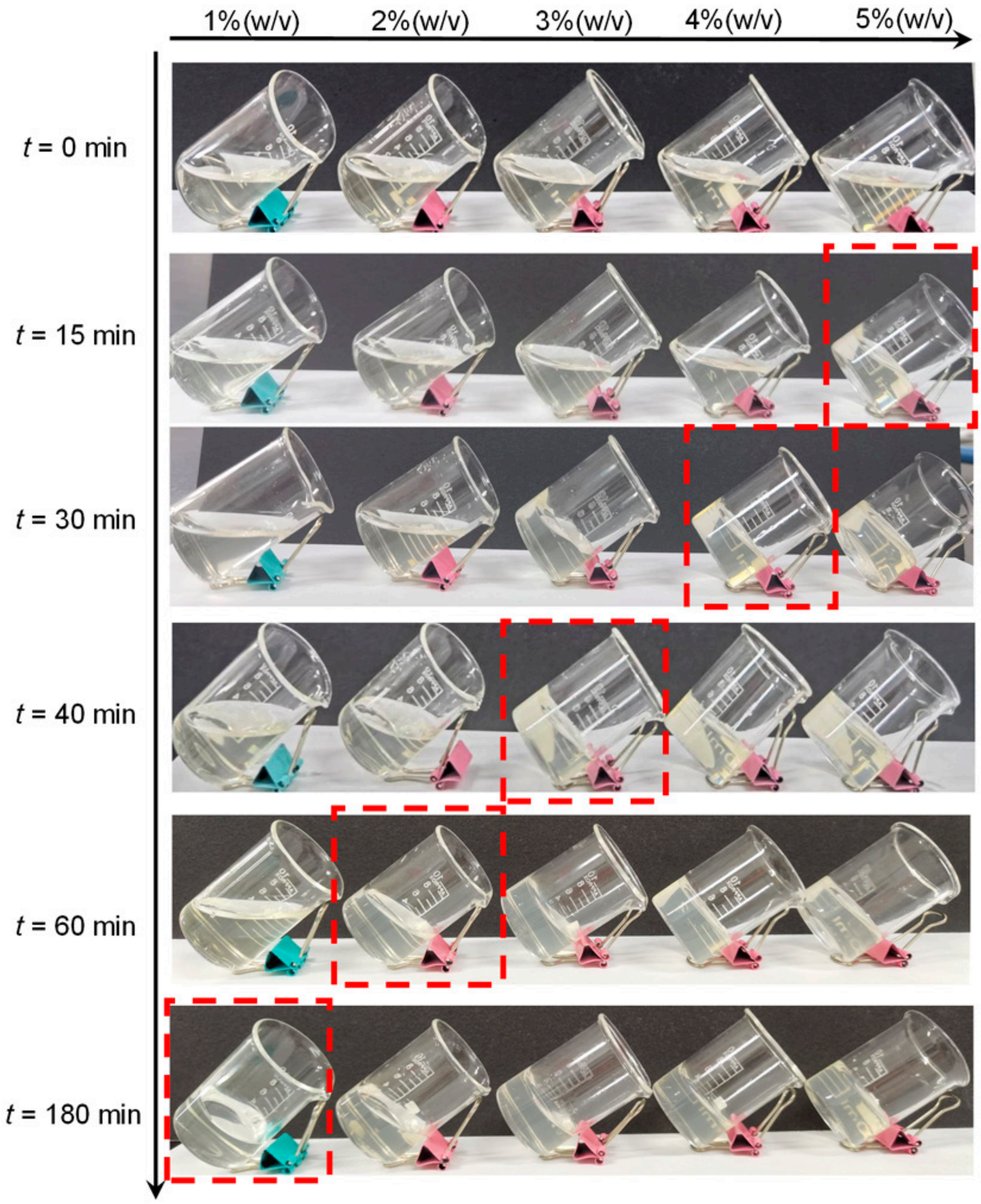

Figure S3. Influence of GDL content on the gelling process of alginate-contained aqueous solution. The sodium alginate and EDTA-Ca are fixed at 2% (w/v) and 0.10 M respectively.

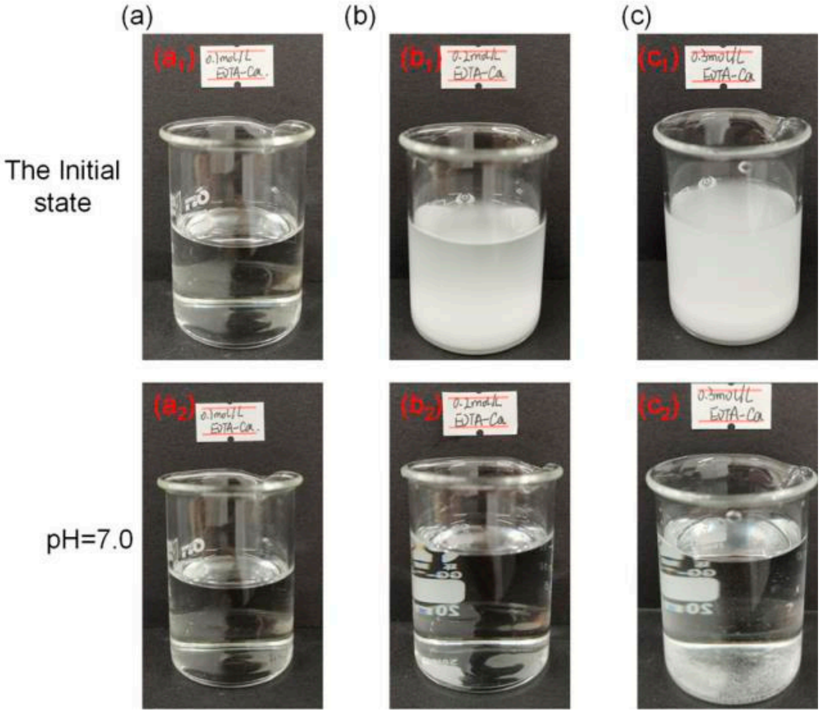

Figure S4. Optical microscope pictures of EDTA-Ca aqueous solution with different concentration.

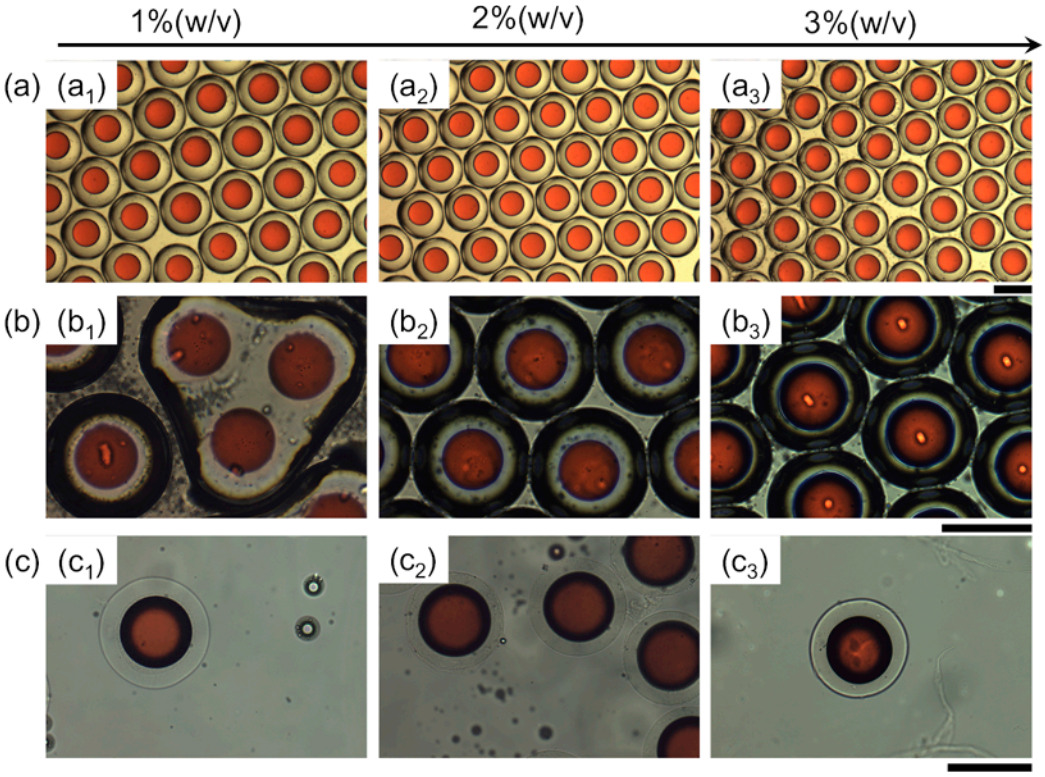

Figure S5. Optical microscope pictures of O/W/O emulsions with varied alginate sodium content in the middle aqueous layer (**a**) and the corresponding alginate

microcapsules in collection solution (**b**) and DI water (**c**). The GDL and EDTA-Ca are fixed at 2.0% (w/v) and 0.10 M respectively. Scale bars are 200 μm.

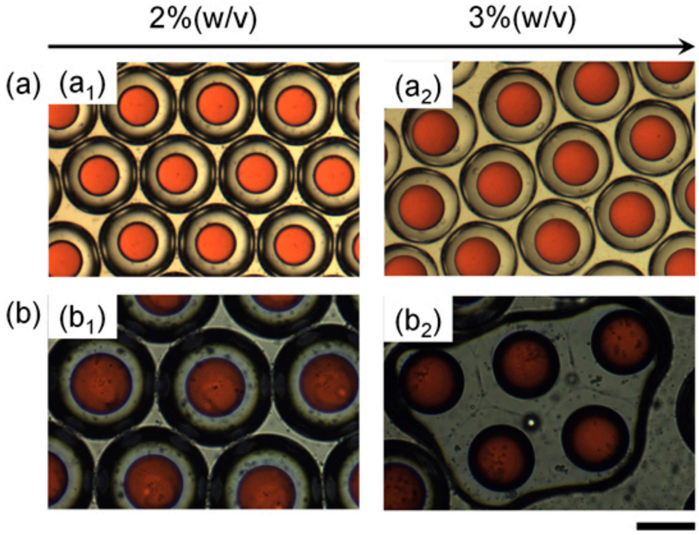

Figure S6 Optical microscope pictures of O/W/O emulsions with varied GDL content in the middle aqueous layer (**a**) and the corresponding alginate microcapsules in collection solution (**b**). The alginate sodium and EDTA-Ca are fixed at 2.0% (w/v) and 0.10 M respectively. Scale bars are 200 μm.

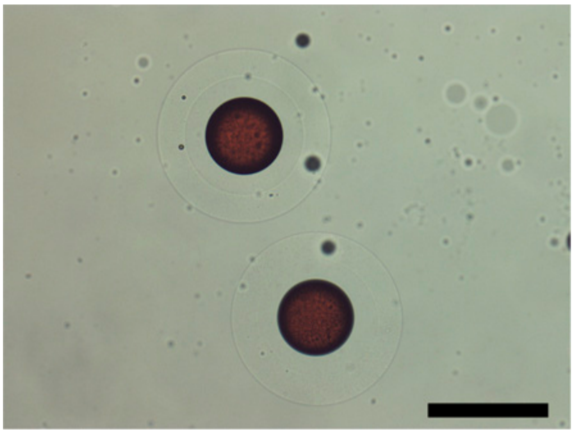

Figure 7 Optical microscope pictures of alginate microcapsules in DI water. Scale bars are 200 μm.

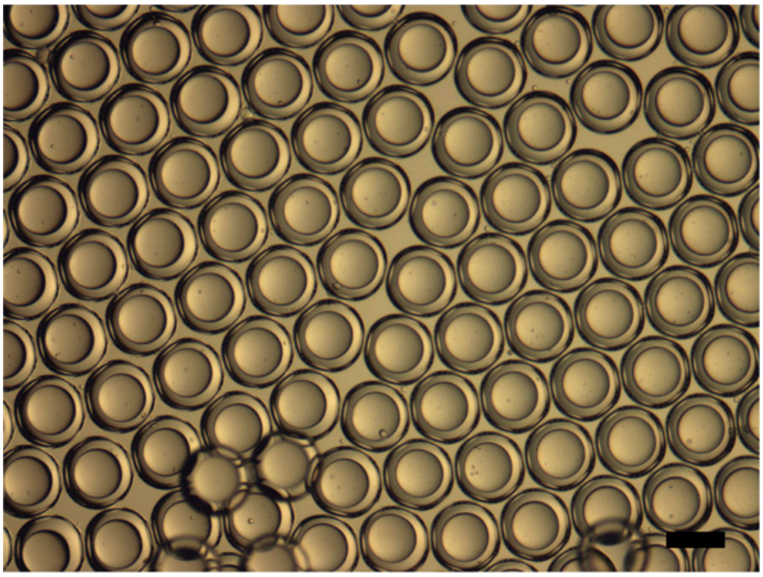

Figure S8. Optical microscope pictures of O/W/O emulsions for citral-contained alginate microcapsules. Scale bars are 200 μm. The average diameters of the O/W/O emulsions and the inner oil droplets are 270 μm (*CV* = 2.8%) and 190 μm (*CV* = 3.1%) respectively.
